# Supplementary material for: Optical blood-brain-tumor barrier modulation expands therapeutic options for glioblastoma treatment
Source: Nat Commun. 2023 Aug 15;14:4934. doi: 10.1038/s41467-023-40579-1 (PMC10427669; doi:10.1038/s41467-023-40579-1)
Supplement: Supplementary file 1 — Supplementary information [file 41467_2023_40579_MOESM1_ESM.pdf]

Supplementary Information

Optical Blood-Brain-Tumor Barrier Modulation Expands Therapeutic Options for Glioblastoma Treatment

Qi Cai[1], Xiaoqing Li[2], Hejian Xiong[1], Hanwen Fan[1], Xiaofei Gao[3], Vamsidhara Vemireddy[3,4], Ryan Margolis[2], Junjie Li[2], Xiaoqian Ge[1], Monica Giannotta[5,9], Kenneth Hoyt[2], Elizabeth Maher[3,4,6], Robert Bachoo[3,4,6]*, Zhenpeng Qin[1,2,7,8]*

[1]Department of Mechanical Engineering, the University of Texas at Dallas, Richardson, TX 75080, USA

[2]Department of Bioengineering, the University of Texas at Dallas, Richardson, TX 75080, USA

[3]Department of Internal Medicine, University of Texas Southwestern Medical Center, Dallas, TX 75390, USA

[4]Harold C. Simmons Comprehensive Cancer Center, University of Texas Southwestern Medical Center, Dallas, TX 75390, USA

[5]IFOM ETS - The AIRC Institute of Molecular Oncology, 20139 Milan, Italy

[6]Department of Neurology, University of Texas Southwestern Medical Center, Dallas, TX 75390, USA

[7]Department of Biomedical Engineering, University of Texas Southwestern Medical Center, Dallas, TX 75390, USA

[8]Center for Advanced Pain Studies, the University of Texas at Dallas, Richardson, TX 75080, USA

[9]Division of Immunology, Transplantation and Infectious Diseases, IRCCS San Raffaele Scientific Institute, 20132 Milan, Italy

This PDF file includes the following:

Supplementary Fig. 1-13

Table S1-S4

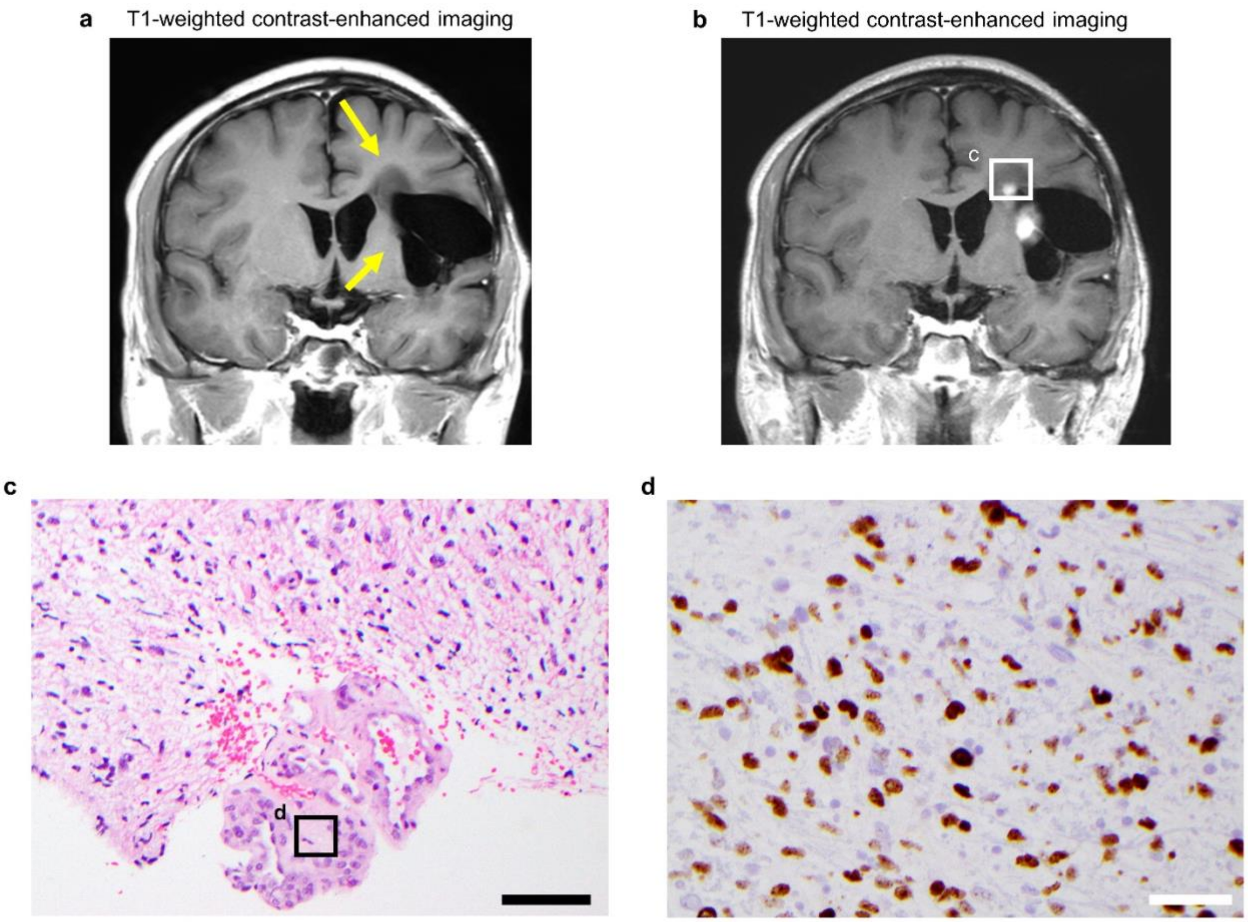

Supplementary Fig. 1 Human GBM shows marginal recurrence associated with no initial contrast enhancement and intratumoral BBTB heterogeneity. a MR image (T1-weighted contrast-enhanced imaging) of a patient with a high-grade glioma who completed the standard treatment, and for 4 years of serial imaging, there was no evidence of recurrence. The yellow arrows show the area of intact BBTB. **b** MR image with gadolinium (T1-weighted contrast-enhanced imaging) of this patient demonstrates the development of new contrast enhancement in the surgical margin. **c, d** Hematoxylin and Eosin (H&E) and MIB-1 staining from resected enhancing tumor in **b** demonstrate a highly proliferative tumor (MIB-1 80%) with microvascular proliferation. No biological replication was performed. The scale bars represent 20 μm and 50 μm in **c** and **d**, respectively.

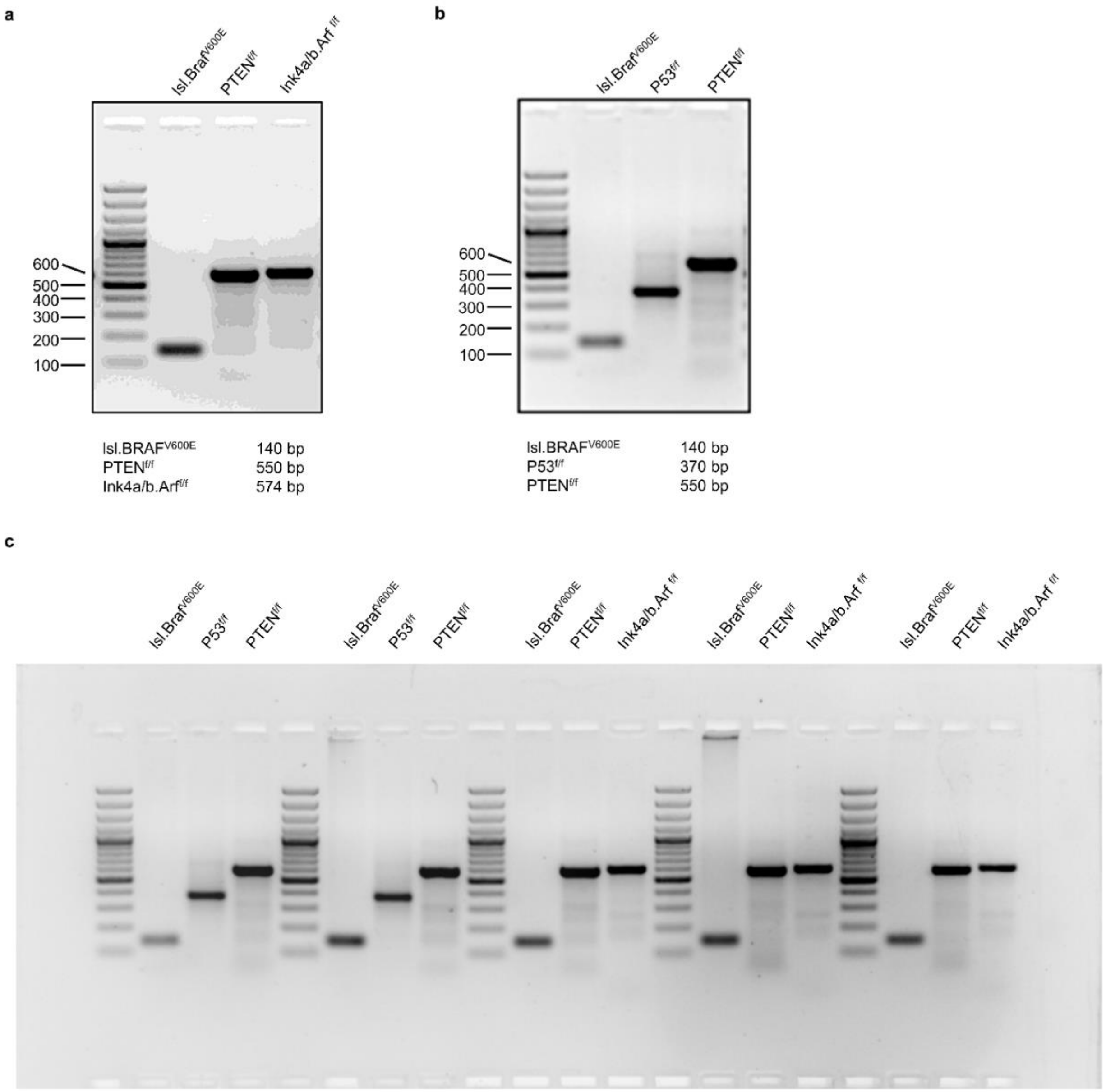

Supplementary Fig. 2. Genotyping of the cell lines by Polymerase chain reaction (PCR). a PS5A1 cell line carried conditional floxed tumor suppressor genes $PTEN^{f/f}$ and $Ink4a/b.Arf^{f/f}$ along with the conditional (lox-stop-lox) $Braf^{V600E\ f/+}$. **b** 73c cell line carried (lox-stop-lox) $Braf^{V600E\ f/+}$, $P53^{f/f}$ and $PTEN^{f/f}$. **c** Uncropped scan of the gel.

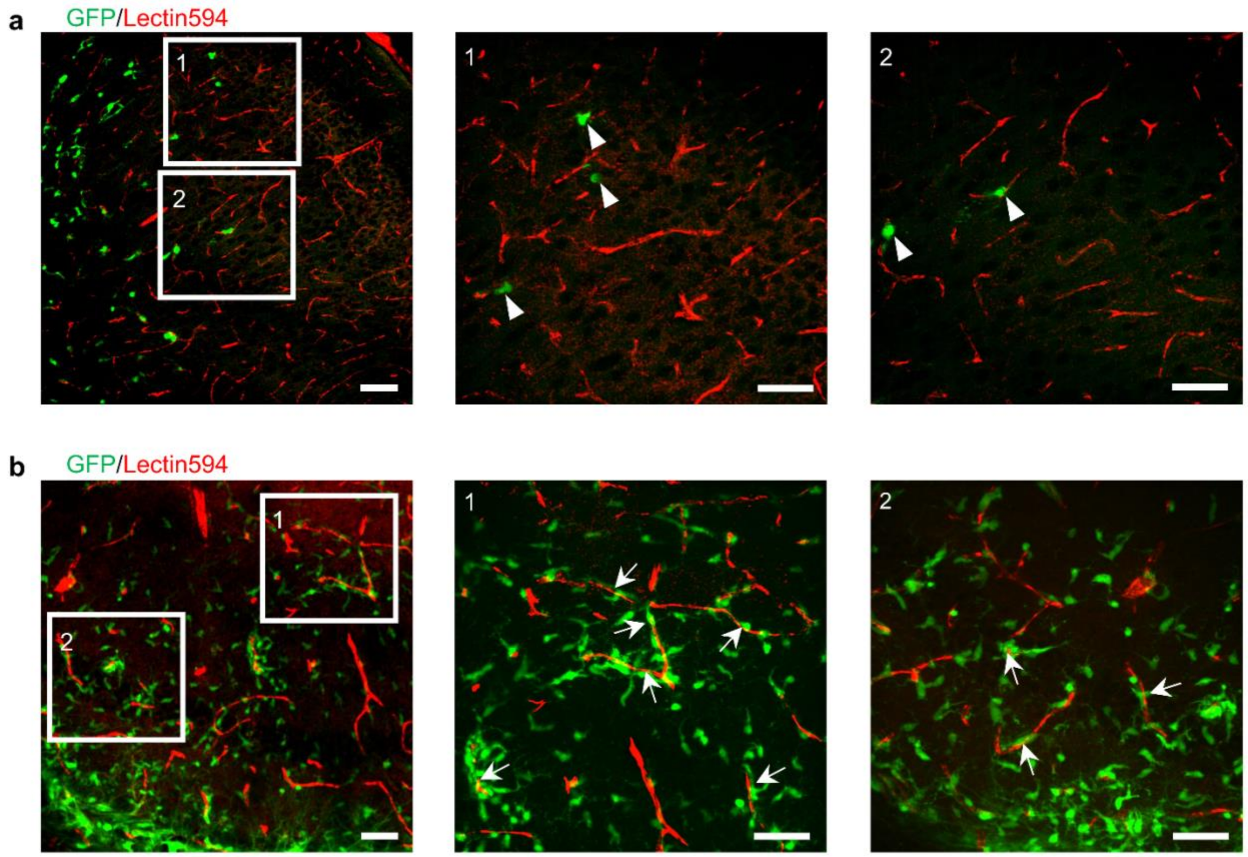

Supplementary Fig. 3 Characterization of PS5A1 GEMM shows single-cell infiltration and vessel co-option growth pattern. a Characterization of the vessel single-cell infiltrative growth pattern (arrowheads). **b** Characterization of the vessel co-option growth pattern (arrows). In **a** and **b**, the blood vessels are labeled with lectin594, and the tumor cells are indicated by GFP. Two independent experiments were performed and similar results are provided in the Source Data file. Scale bars represent 50 µm.

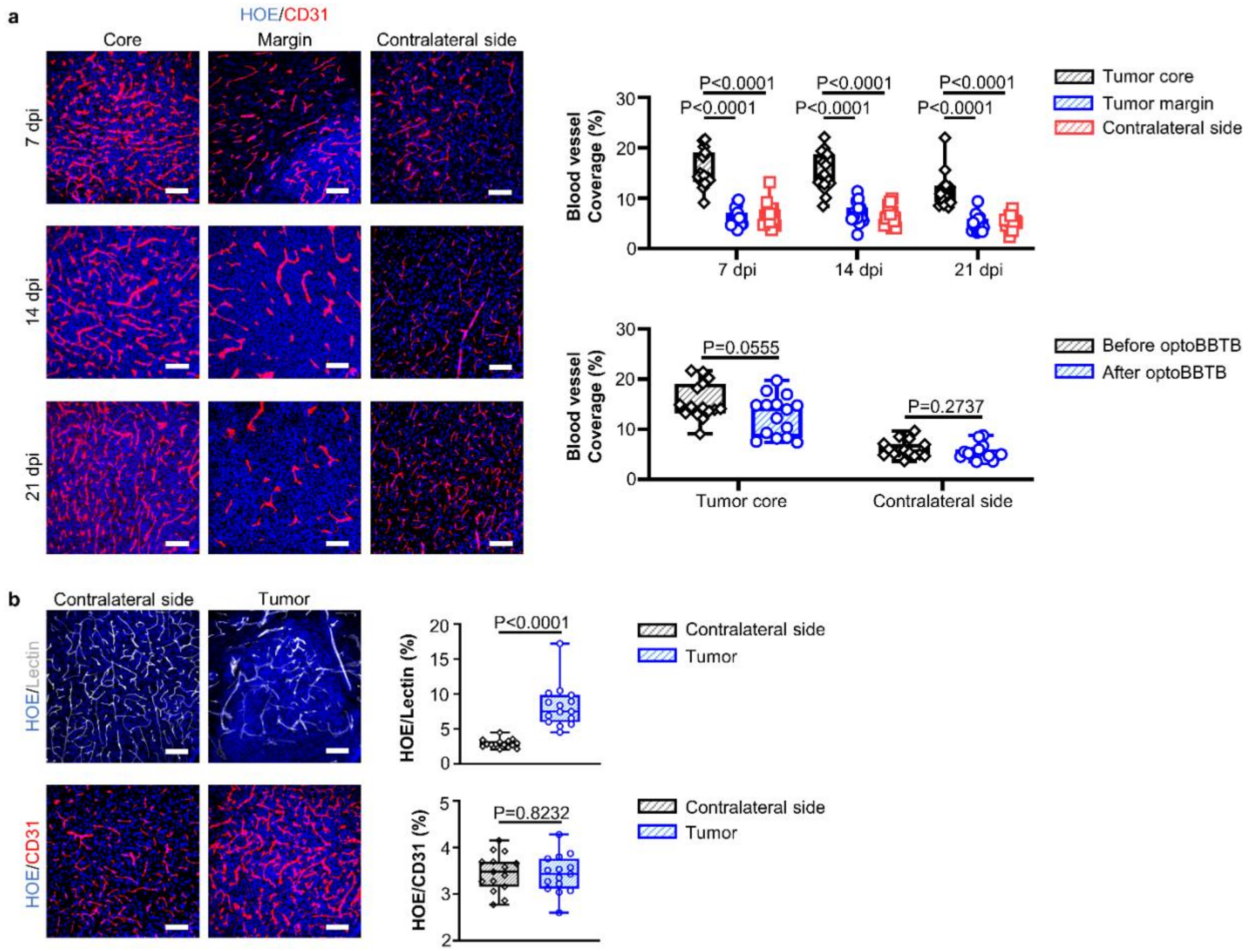

Supplementary Fig. 4 Blood vessel labeling in 73C GEMM shows denser but not well-perfused vasculature. a IHC staining and quantification of blood vessels using CD31 at 7-21 dpi (days post injection). The cell nuclei are labeled with Hoechst staining (HOE). The scale bars represent 100 µm. Quantification of blood vessel coverage was performed by analyzing CD31 area fraction. N=15 images from 3 mice. **b** A comparison of blood vessels labeling with tomato lectin594 or CD31 at 7 dpi. The cell nuclei are labeled by Hoechst staining (HOE). The scale bars represent 100 µm. The ratio of cell nuclei to blood vessels was quantified by area fraction. N=15 images from 3 mice. Data in the box and whisker plots are given from the minima to maxima, the bounds of the box represent the 25th percentile and 75th percentile, and the middle line of the box is the median. Data were analyzed by One-way ANOVA followed by Tukey's multiple

comparisons test in **a** and unpaired Student's two-sided *t* test in **b**. Source data are available as a Source Data file.

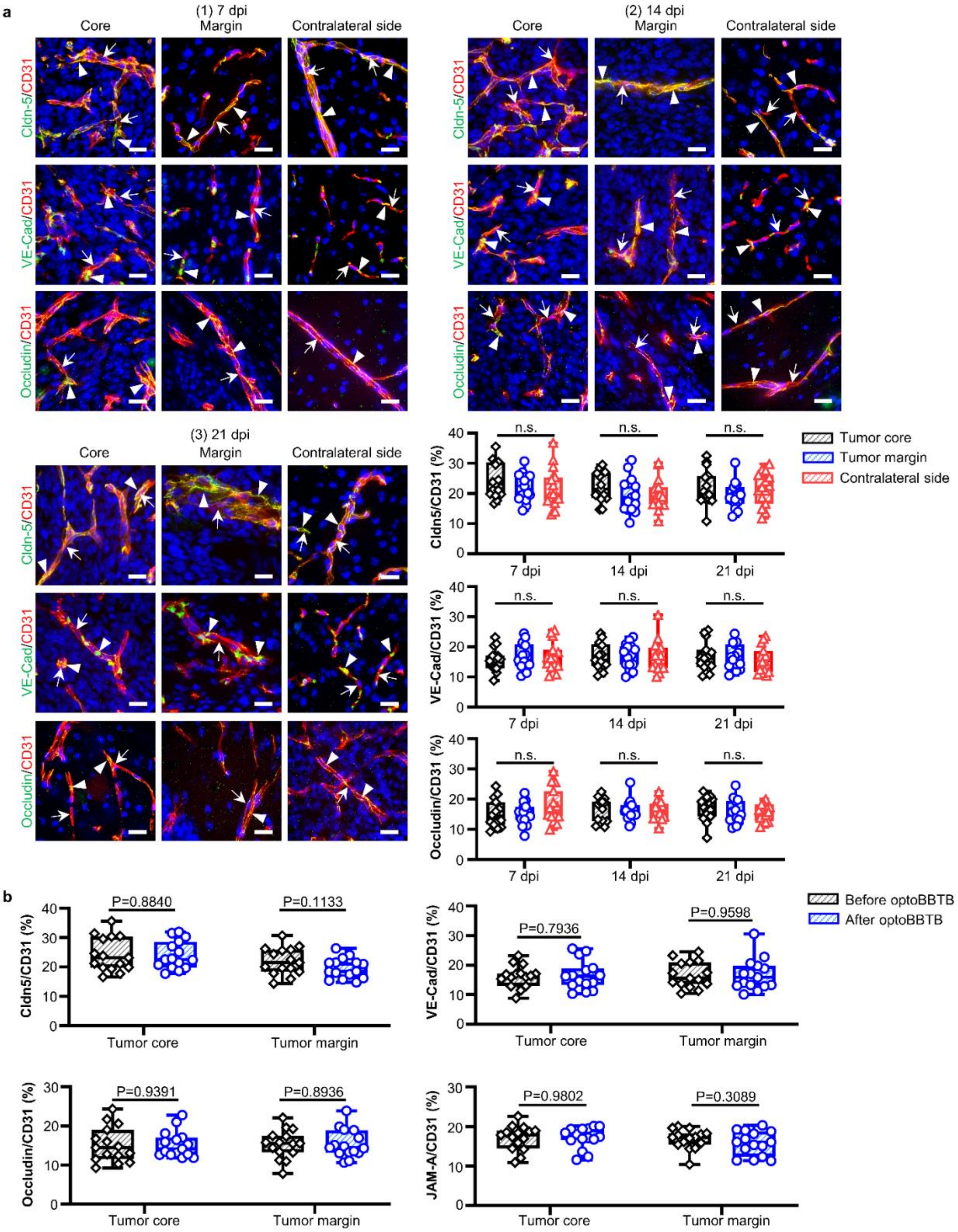
a
(1) 7 dpi
Core
Margin
Contralateral side
(2) 14 dpi
Core
Margin
Contralateral side
Cldn-5/CD31
VE-Cad/CD31
Occludin/CD31
(3) 21 dpi
Core
Margin
Contralateral side
Cldn-5/CD31
VE-Cad/CD31
Occludin/CD31
Tumor core
Tumor margin
Contralateral side
Cldn5/CD31 (%)
VE-Cad/CD31 (%)
Occludin/CD31 (%)
n.s.
7 dpi
14 dpi
21 dpi
b
Before optoBBTB
After optoBBTB
Cldn5/CD31 (%)
P=0.8840
P=0.1133
VE-Cad/CD31 (%)
P=0.7936
P=0.9598
Occludin/CD31 (%)
P=0.9391
P=0.8936
JAM-A/CD31 (%)
P=0.9802
P=0.3089
Tumor core
Tumor margin

Supplementary Fig. 5 Junctional proteins labeling in 73C GEMM shows no significant changes in Claudin-5, VE-Cadherin, or Occludin. a IHC staining of Claudin-5 (Cldn5), VE-Cadherin (VE-Cad), and Occludin at 7-21 dpi. The blood vessels are stained with CD31, and the cell nuclei are indicated by Hoechst staining (HOE). The arrow indicates blood vessels, and the arrowhead indicates junctional proteins. The scale bars represent 20 µm. Quantification analysis of the expression of Claudin-5, VE-Cadherin, and Occludin over CD31 was performed by area fraction. N=15 images from 3 mice. **b** The quantification of the expression of junctional proteins over CD31 before and after optoBBTB was performed by analyzing area fraction. N=15 images from 3 mice. Data in the box and whisker plots are given from the minima to maxima, the bounds of the box represent the 25th percentile and 75th percentile, and the middle line of the box is the median. Data were analyzed by One-way ANOVA followed by Tukey's multiple comparisons test in **a** or unpaired Student's two-sided *t* test in **b**. Source data are available as a Source Data file.

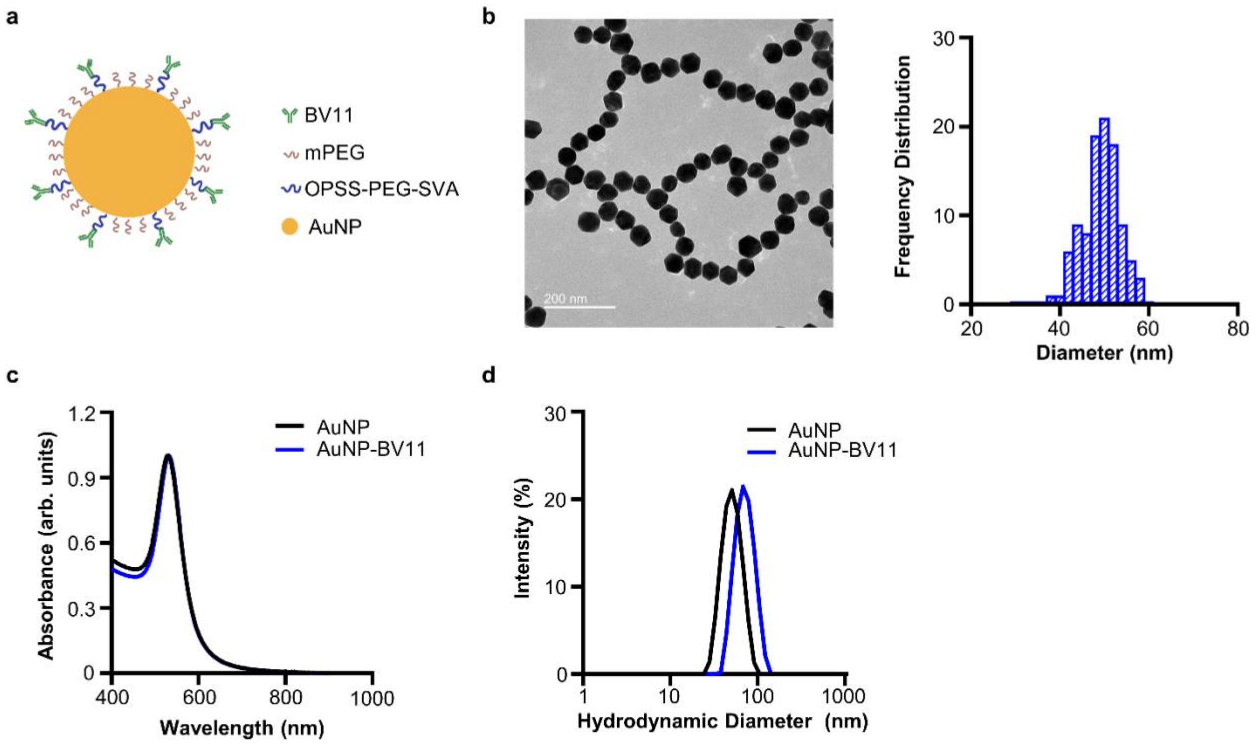

Supplementary Fig. 6 Characterization of AuNP-BV11. a The surface functionalization of AuNP-BV11. The illustration figure was created with Biorender.com. **b** The morphology and size of the AuNP core are characterized by Transmission Electron Microscopy. The size of the nanoparticles (50 ± 4 nm) was measured with Image-J by manually counting 100 particles. **c** The localized surface plasmon resonance peak of the nanoparticles is characterized by UV-Vis-NIR spectroscopy. **d** The nanoparticle hydrodynamic diameter distribution by relative intensity is characterized by Dynamic Light Scattering. The Z-average for AuNP and AuNP-BV11 was 49 nm and 69 nm, respectively. Each batch of the AuNP-BV11 was characterized before using, and a representative set of characterizations is shown in the figure. Source data are available as a Source Data file.

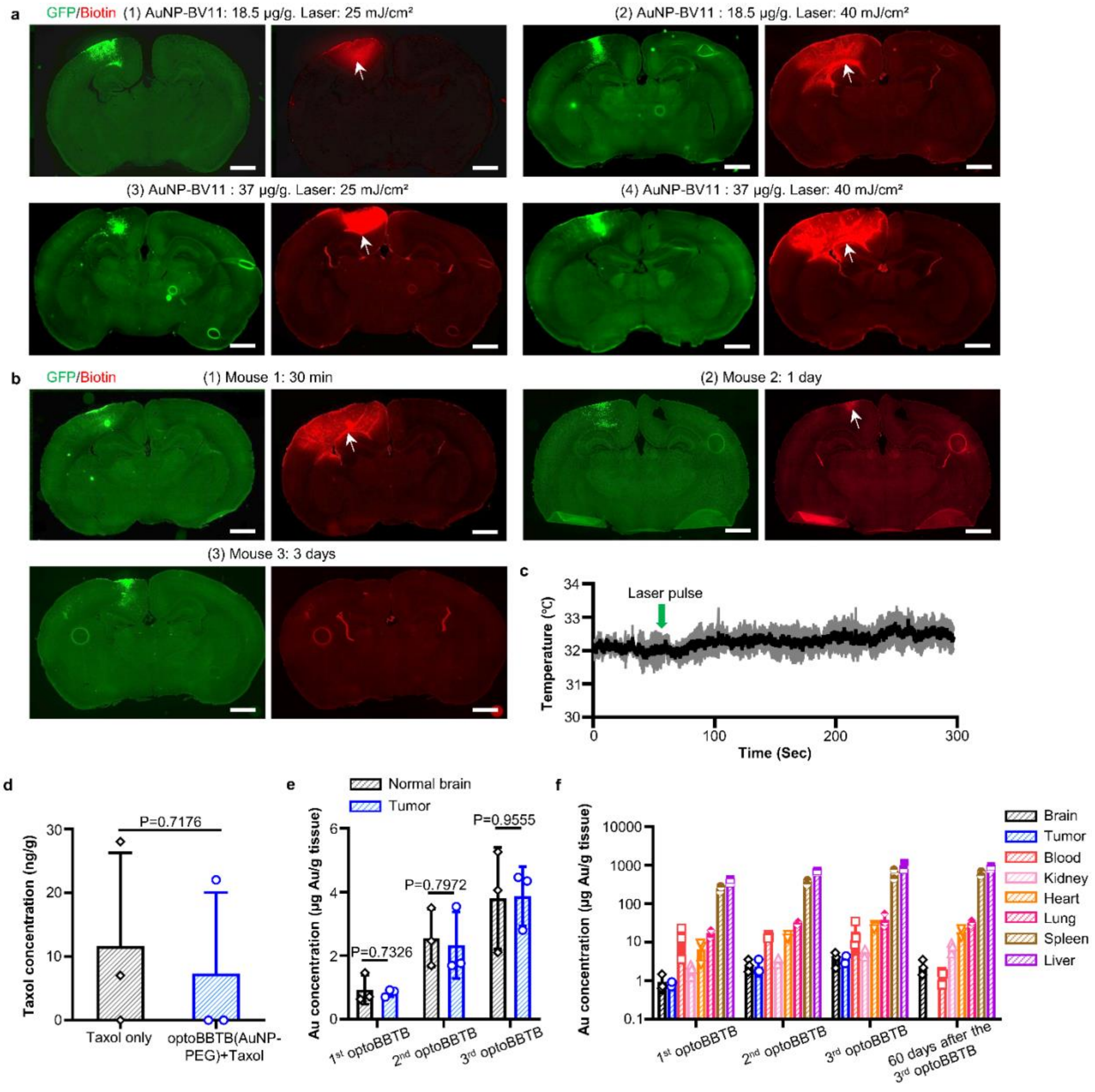

Supplementary Fig. 7 BBTB modulation in PS5A1 GEMM. a Optimization of optoBBTB in PS5A1 GEMM. AuNP-BV11 injection dose was 18.5 μg/g or 37 μg/g. Laser fluence was 25 mJ/cm^2 or 40 mJ/cm^2. The pulse number was 1. The dye leakage is indicated by arrows. The scale bar represents 1 mm. **b** Recovery of BBTB permeability after optoBBTB at 30 min, 1 day, and 3 days. Tumor cells are indicated by GFP, and the biotin leakage is indicated by arrows. The scale bar represents 1 mm. **c** The record of temperature change after optoBBTB. The AuNP-BV11

injection dose was 18.5 μg/g, and the laser fluence was 40 mJ/cm^2 (1 pulse). N=3 mice, data are expressed as Mean ± SD. **d** The analysis of Taxol concentration in PS5A1 GEMM under conditions (1) Taxol administration into the PS5A1 GEMM (Taxol only, 14 days post injection, dpi), and (2) optoBBTB using AuNP-PEG followed by Taxol administration at 14 dpi. N=3 mice. Data are expressed as Mean ± SD. **e** The analysis of the AuNP-BV11 accumulation in the normal brain and the tumor after each optoBBTB. N=3 mice, data are expressed as Mean ± SD. **f** The biodistribution of AuNP-BV11 in PS5A1 GEMM after each optoBBTB and the nanoparticle degradation analysis. N=3 mice, data are expressed as Mean ± SD. Data were analyzed by unpaired Student's two-sided *t* test in **d** and **e**. In **a** and **b**, two independent experiments were performed and similar results are provided in the Source Data file. Source data are available as a Source Data file.

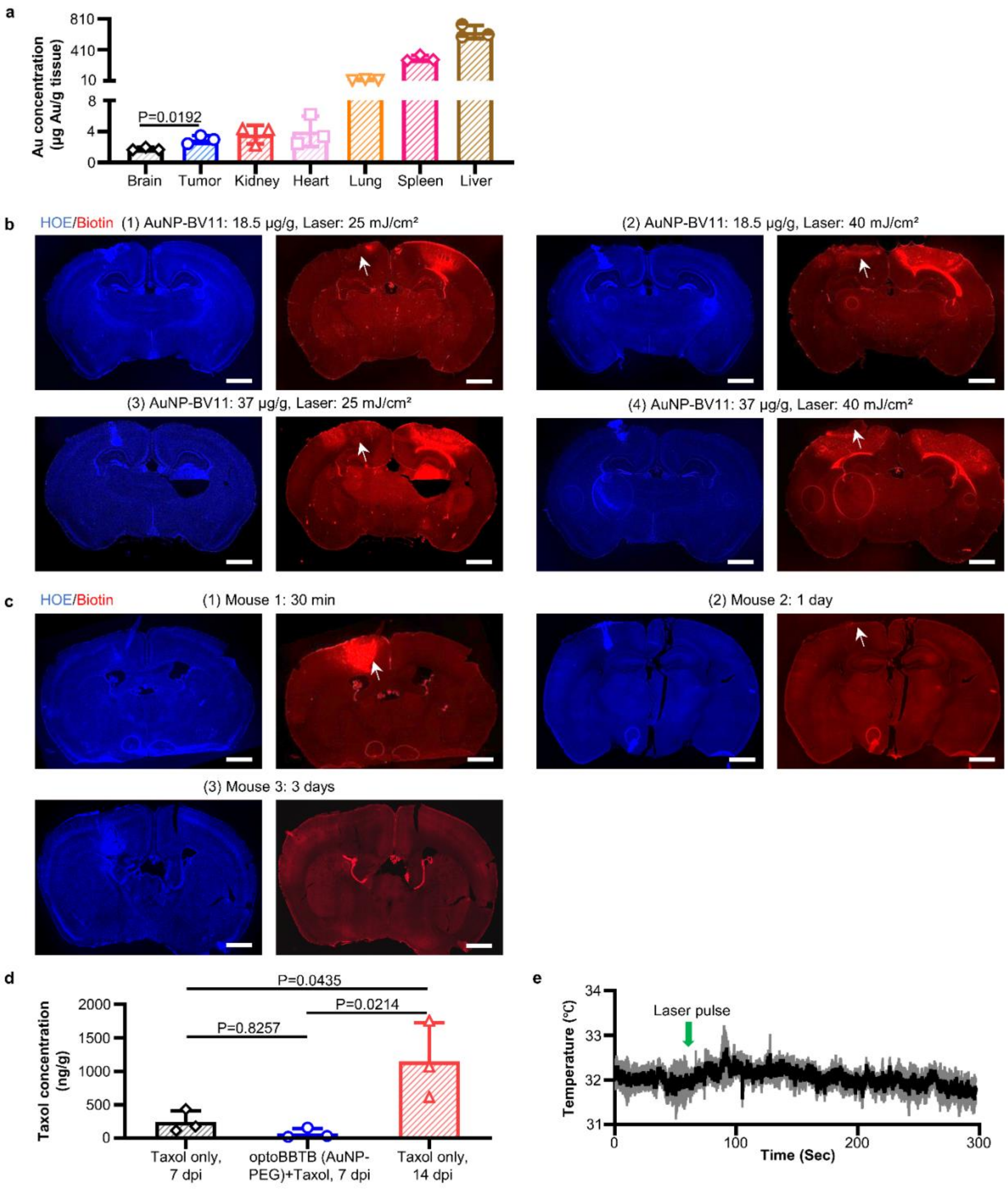

Supplementary Fig. 8 BBTB modulation in 73C GEMM. a The biodistribution of AuNP-BV11 in 73C GEMM at 7 days post injection (dpi). The AuNP-BV11 injection dose was 37 µg/g. N=3 mice, data are expressed as Mean ± SD. **b** Optimization of optoBBTB in 73C GEMM at 7

dpi. AuNP-BV11 injection dose was 18.5 μg/g or 37 μg/g. Laser fluence was 25 mJ/cm^2 or 40 mJ/cm^2. The pulse number was 1. A laser pulse was applied to both sides of the brain. Biotin leakage is indicated by arrows. Cell nuclei are indicated by Hoechst staining (HOE). The scale bar represents 1 mm. **c** Recovery of BBTB permeability after optoBBTB at 30 min, 1 day, and 3 days. Biotin leakage is indicated by arrows. Cell nuclei are indicated by Hoechst staining (HOE). The scale bar represents 1 mm. **d** The analysis of Taxol concentration in 73C GEMM under conditions (1) Taxol administration into the 73C GEMM at 7 dpi (Taxol only, 7 dpi), and (2) optoBBTB using AuNP-PEG followed by Taxol administration at 7 dpi, and (3) Taxol administration into the 73C GEMM at 14 dpi (Taxol only, 14 dpi). N=3 mice. Data are expressed as Mean ± SD. **e** Temperature change after optoBBTB. The AuNP-BV11 injection dose was 37 μg/g, and the laser fluence was 40 mJ/cm^2 (1 pulse). The data are expressed as Mean ± SD. N=3 mice. Data were analyzed by unpaired Student's two-sided *t* test in **a** and One-way ANOVA followed by Tukey's multiple comparisons test in **d**. In **b** and **c**, two independent experiments were performed, similar results are provided in the Source Data file. Source data are available as a Source Data file.

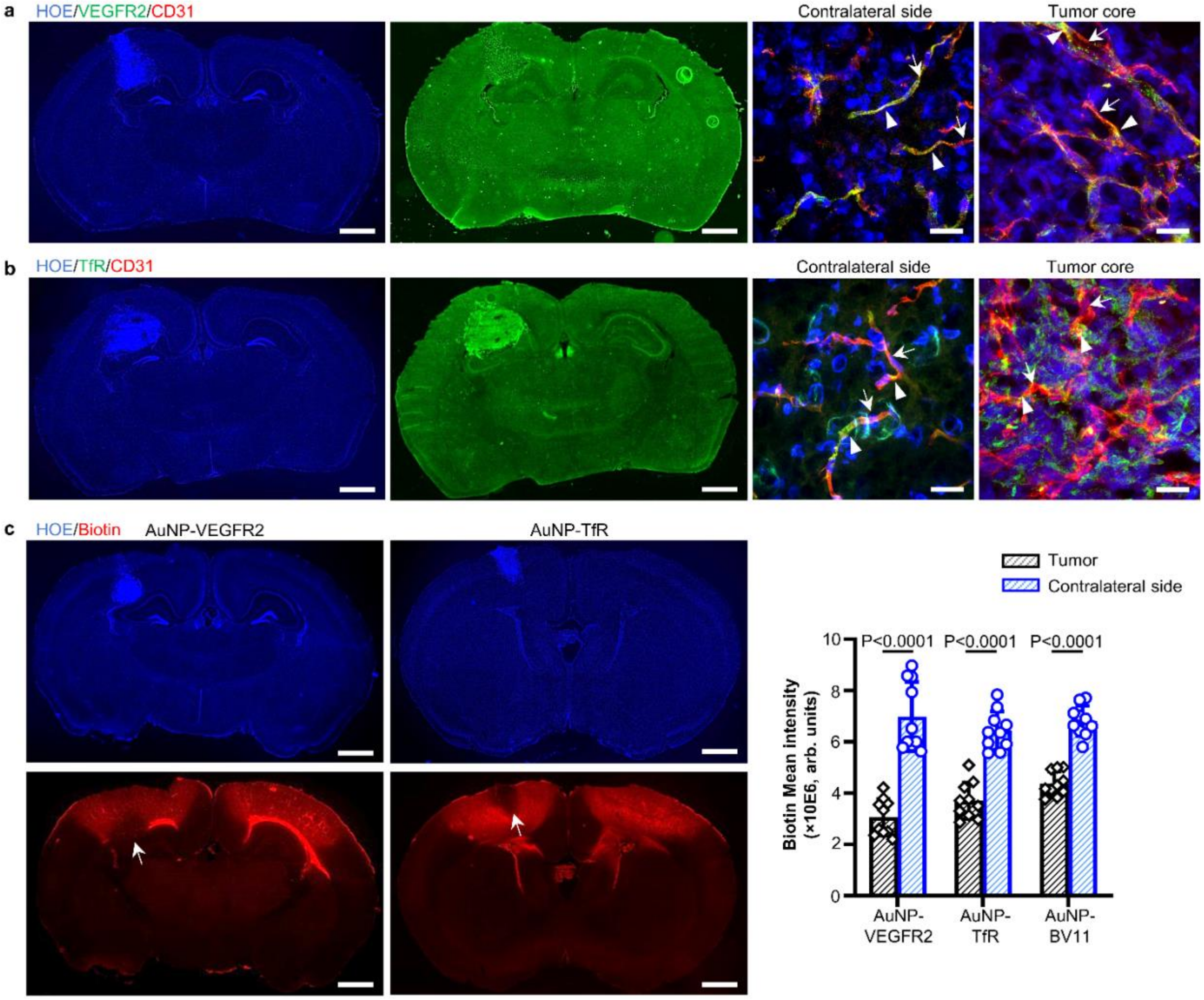

Supplementary Fig. 9 BBTB modulation using different targets in the 73C GEMM. a IHC staining shows the overexpression of vascular endothelial growth factor receptor 2 (VEGFR2) at 7 days post injection (dpi). The blood vessels are stained by CD31. The cell nuclei are indicated by Hoechst staining (HOE). **b** IHC staining shows the overexpression of transferrin receptor (TfR) at 7 dpi. The blood vessels are stained by CD31. The cell nuclei are indicated by Hoechst staining (HOE). **c** A comparison of BBTB modulation efficacy using AuNP-VEGFR2 and AuNP-TfR. The cell nuclei are indicated by Hoechst staining (HOE). The biotin leakage is indicated by arrows. The nanoparticle dose is 37 μg/g, and the laser fluence is 40 mJ/cm^2, 1 pulse. A laser pulse was applied to both sides of the brain. The tumor was injected into the left side of the brain,

and the right side served as an internal control. The optoBBTB efficiency was quantified by analyzing the biotin mean fluorescent intensity in the tumor core and the contralateral. Data are expressed as Mean ± SD, N=10 images from 3 mice. Data were analyzed by unpaired Student's two-sided *t* test. The scale bars represent 1 mm in the slide scanner images in **a-c** and 20 µm in zoom-in images in **a** and **b**. In **a** and **b**, two independent experiments were performed, similar results are provided in the Source Data file. Source data are available as a Source Data file.

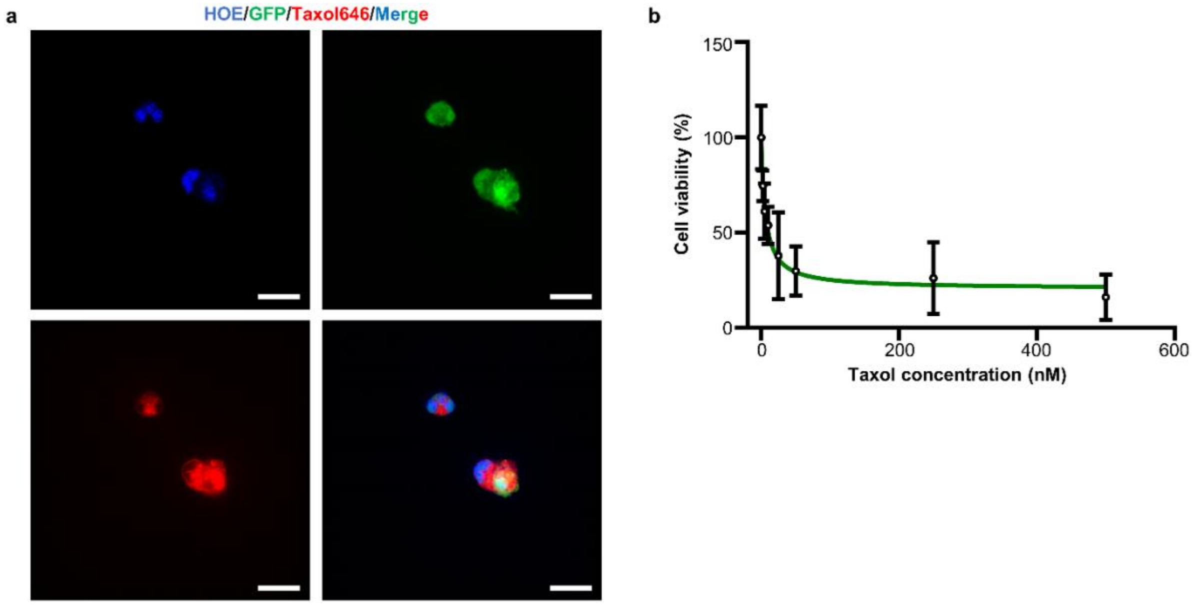

Supplementary Fig. 10 In vitro cellular uptake and cytotoxicity of Taxol in PS5A1 cells. a In vitro cellular uptake of fluorescent Taxol646. Cell nuclei, tumor cells, and Taxol646 are indicated by Hoechst staining (HOE), green color (GFP), and red color, respectively. The scale bars represent 20 μm. **b** Cell viability of PS5A1 cells at various concentrations (0 to 500 nM) of Taxol. The IC_{50} value at 72 hours is 6.3 nM. Data are expressed as Mean ± SD, N=6 replicates. Source data are available as a Source Data file.

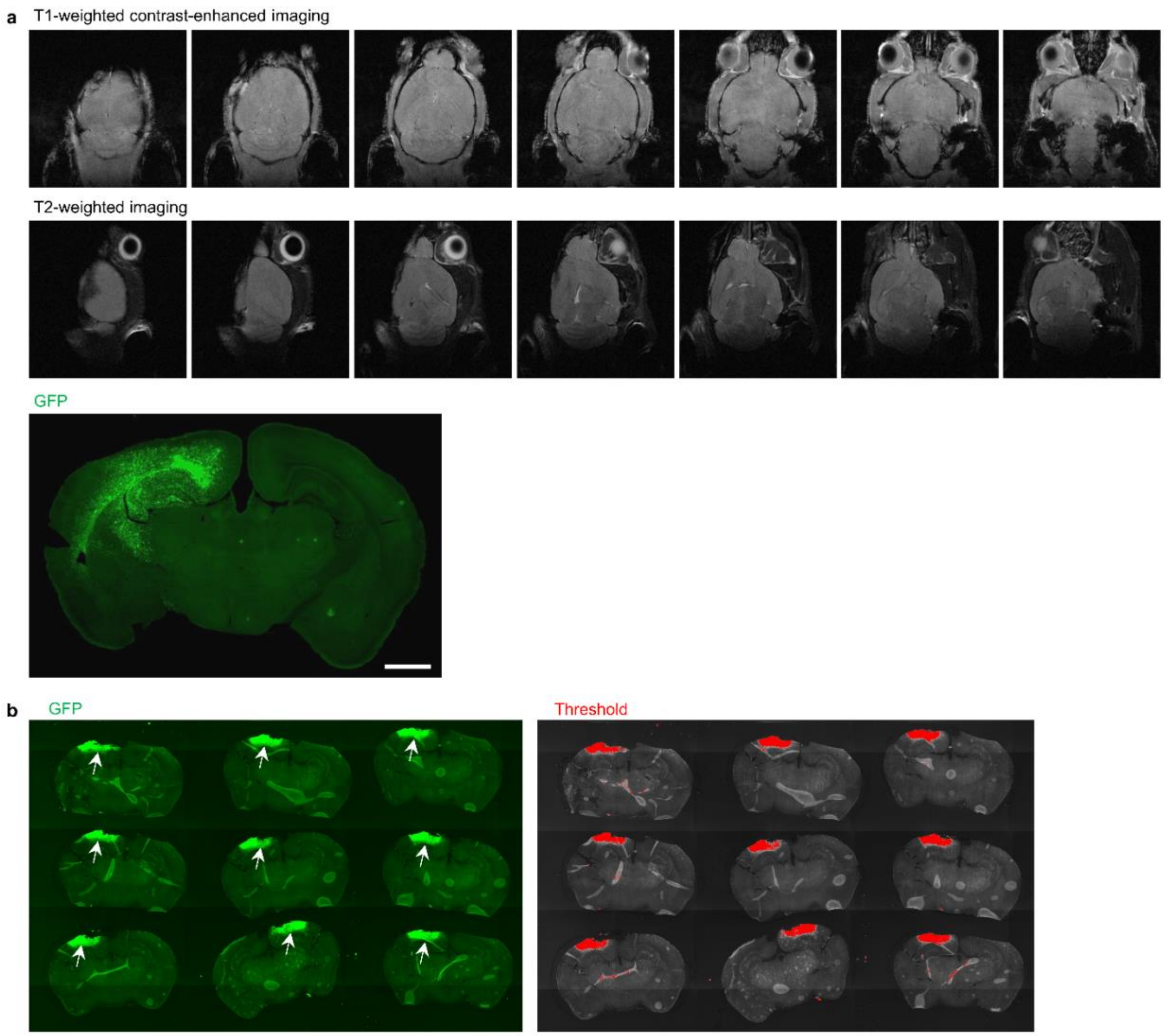

Supplementary Fig. 11 Measurement of PS5A1 tumor size. a Magnetic Resonance Imaging of the tumor at 42 days post injection (dpi) shows no T1-weighted contrast enhancement or T2-weighted hyperintensity, while fluorescent imaging of GFP confirmed the presence of the tumor. The scale bar represents 1 mm. No biological replication was performed. **b** Tumor volume analysis using fluorescent images. Left: an example of the original fluorescent image. The tumors are indicated by GFP fluorescent and arrows. Right: the image after processing with the threshold function in Fiji/Image-J.

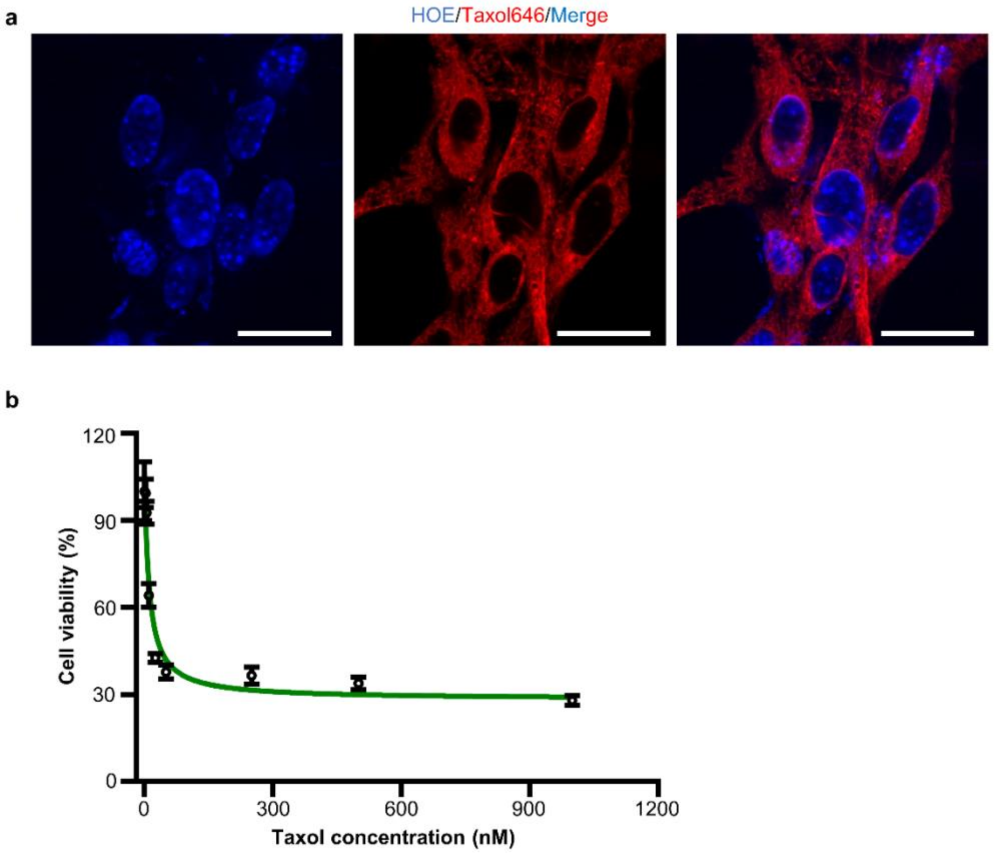

Supplementary Fig. 12 In vitro cellular uptake and cytotoxicity of Taxol646 in 73C tumor cells. a In vitro cellular uptake of fluorescent Taxol646. Cell nuclei and Taxol646 are indicated by Hoechst staining (HOE) and red color, respectively. The scale bars represent 10 µm. **b** Cell viability of 73C tumor cells at various concentrations (0 to 1000 nM) of Taxol. The IC_{50} value at 72 hours is 10.52 nM. Data are expressed as Mean ± SD, N=6 replicates. Source data are available as a Source Data file.

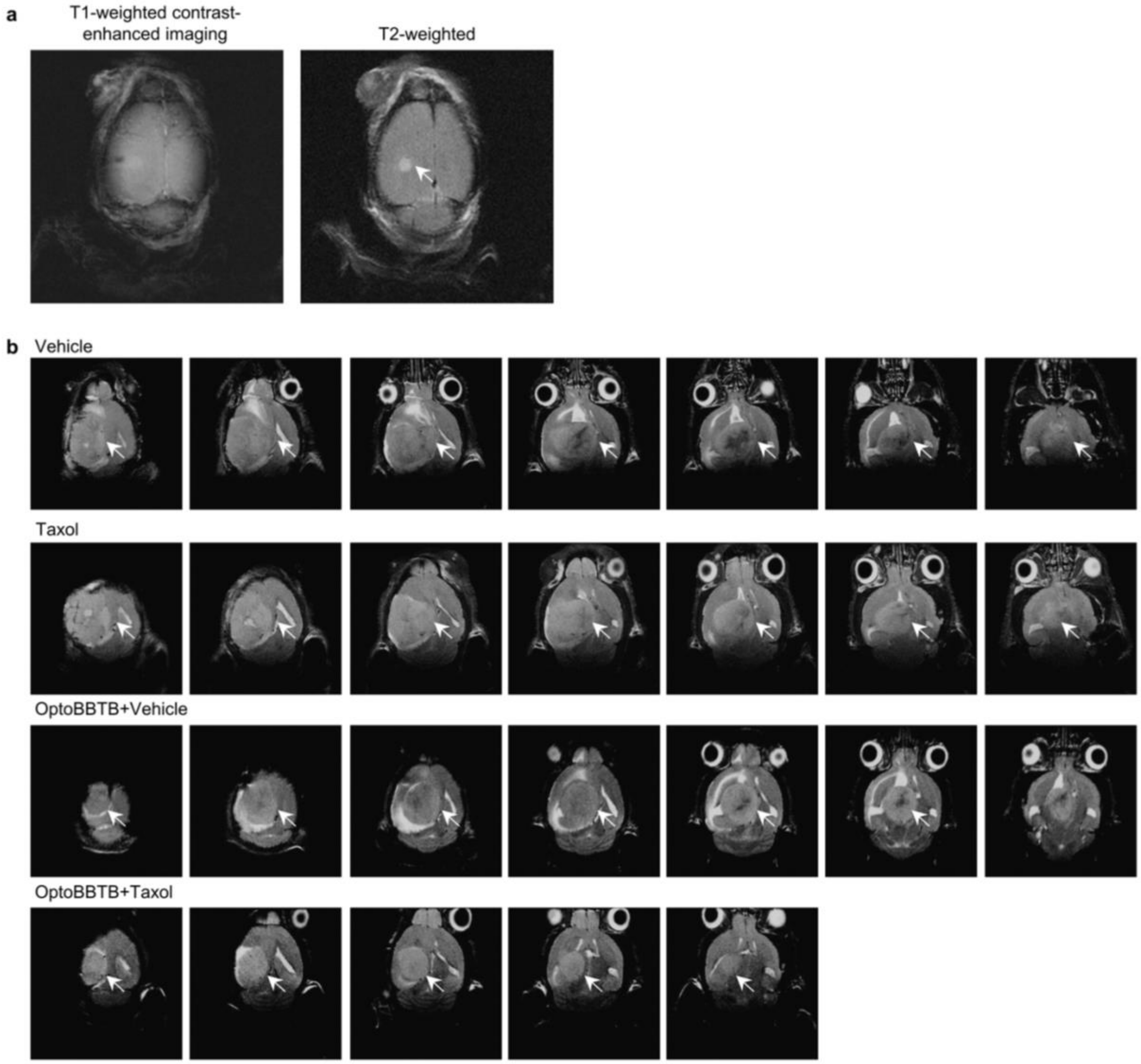

Supplementary Fig. 13 Tumor size analysis of 73C GEMM by MRI. a Magnetic Resonance Imaging of 73C GBM at 3 dpi. Top, T1-weighted scan post gadolinium injection. Bottom, T2-weighted scan. **b** Representative Magnetic resonance imaging of the tumor volume in each group at 15 dpi after 3 treatments. Representative images were from 1 mouse in each group. The tumors were indicated by arrows.

Supplementary Table 1. Optimization of the optoBBTB in PS5A1 GEMM.

	Nanoparticle type	Nanoparticle dose	Laser dose
Condition 1	AuNP-BV11	18.5 µg/g	25 mJ/cm^2, 1 pulse
Condition 2	AuNP-BV11	18.5 µg/g	40 mJ/cm^2, 1 pulse
Condition 3	AuNP-BV11	37 µg/g	25 mJ/cm^2, 1 pulse
Condition 4	AuNP-BV11	37 µg/g	40 mJ/cm^2, 1 pulse

Supplementary Table 2. Gold concentration in PS5A1 GEMM after three optoBBTB. Unit: µg (Au)/g (tissue).

	Brain	Tumor	Blood	Kidney	Heart	Lung	Spleen	Liver
1st optoBBTB	0.9 ± 0.5	1.3 ± 0.4	13 ± 9	2.0 ± 0.7	6 ± 3	18 ± 4	250 ± 40	370 ± 50
2nd optoBBTB	2.6 ± 0.9	2 ± 1	13.9 ± 1.4	3.4 ± 0.6	13.5 ± 1.8	32 ± 3	370 ± 50	670 ± 30
3rd optoBBTB	4 ± 1	3.6 ± 1.3	19 ± 14	5.4 ± 1.2	25 ± 3	38 ± 16	610 ± 160	960 ± 180
60 days after the 3rd optoBBTB	2.3 ± 1.1	N/A	1.3 ± 0.5	7.4 ± 2.2	18 ± 4	32 ± 5	620 ± 80	840 ± 70

Supplementary Table 3. Optimization of the optoBBTB in 73C GEMM.

	Nanoparticle type	Nanoparticle dose	Laser dose
Condition 1	AuNP-BV11	18.5 µg/g	25 mJ/cm^2, 1 pulse
Condition 2	AuNP-BV11	18.5 µg/g	40 mJ/cm^2, 1 pulse
Condition 3	AuNP-BV11	37 µg/g	25 mJ/cm^2, 1 pulse
Condition 4	AuNP-BV11	37 µg/g	40 mJ/cm^2, 1 pulse
Condition 5	AuNP-VEGFR2	37 µg/g	40 mJ/cm^2, 1 pulse
Condition 6	AuNP-TfR	37 µg/g	40 mJ/cm^2, 1 pulse

Supplementary Table 4. A comparison of gold concentration in PS5A1 and 73C GEMM. Unit: µg (Au)/g (tissue).

	Brain	Tumor	Kidney	Heart	Lung	Spleen	Liver
PS5A1	0.9 ± 0.5	1.3 ± 0.4	2.0 ± 0.7	6 ± 3	18 ± 4	250 ± 40	370 ± 50
73C	1.8 ± 0.2	3.0 ± 0.5	3.6 ± 1.2	4 ± 2	21.0 ± 7	300 ± 40	640 ± 80
