# Supplementary material for: Optical blood-brain-tumor barrier modulation expands therapeutic options for glioblastoma treatment
Source: Nat Commun. 2023 Aug 15;14:4934. doi: 10.1038/s41467-023-40579-1 (PMC10427669; doi:10.1038/s41467-023-40579-1)
Supplement: Supplementary file 3 — Reporting Summary [file 41467_2023_40579_MOESM3_ESM.pdf]

nature portfolio

Corresponding author(s): Robert Bachoo, Zhenpeng Qin

Last updated by author(s): Jul 27, 2023

Reporting Summary

Nature Portfolio wishes to improve the reproducibility of the work that we publish. This form provides structure for consistency and transparency in reporting. For further information on Nature Portfolio policies, see our Editorial Policies and the Editorial Policy Checklist.

Statistics

For all statistical analyses, confirm that the following items are present in the figure legend, table legend, main text, or Methods section.

n/a	Confirmed	
☐	☒	The exact sample size (*n*) for each experimental group/condition, given as a discrete number and unit of measurement
☐	☒	A statement on whether measurements were taken from distinct samples or whether the same sample was measured repeatedly
☐	☒	The statistical test(s) used AND whether they are one- or two-sided<br>*Only common tests should be described solely by name; describe more complex techniques in the Methods section.*
☒	☐	A description of all covariates tested
☒	☐	A description of any assumptions or corrections, such as tests of normality and adjustment for multiple comparisons
☐	☒	A full description of the statistical parameters including central tendency (e.g. means) or other basic estimates (e.g. regression coefficient) AND variation (e.g. standard deviation) or associated estimates of uncertainty (e.g. confidence intervals)
☐	☒	For null hypothesis testing, the test statistic (e.g. *F*, *t*, *r*) with confidence intervals, effect sizes, degrees of freedom and *P* value noted<br>*Give P values as exact values whenever suitable.*
☒	☐	For Bayesian analysis, information on the choice of priors and Markov chain Monte Carlo settings
☒	☐	For hierarchical and complex designs, identification of the appropriate level for tests and full reporting of outcomes
☒	☐	Estimates of effect sizes (e.g. Cohen's *d*, Pearson's *r*), indicating how they were calculated

Our web collection on statistics for biologists contains articles on many of the points above.

Software and code

Policy information about availability of computer code

Data collection	Fluorescent images: Metamorph software (Version 7.10.1.161) designed for Olympus SD-OSR spinning disk confocal microscope, Olympus VS-ASW (Version 2.9.2) for Olympus VS120 virtual slide scanner, and Living Image® Software (Version 4.7.3) for IVIS® Lumina III In Vivo Imaging System - PerkinElmer.<br>UV-Vis-NIR spectrum: Gen5 (Version 3.11) software for BioTek Synergy 2 plate reader.<br>Nanoparticle size distribution: Malvern Zetasizer software (Version 8.00.4813) for Malvern Zetasizer Nano ZS, and Gatan DigitalMicrograph (Version 3.42.3048.0) for JEOL JEM 1400+ TEM.<br>ICP-MS data: ICP-MS MassHunter Software (4.6 Version C.01.06) for Agilent 7900.<br>Mouse MRI: Paravision 360 (Version 2.0) for BioSpec 3T MRI.
Data analysis	Data were analyzed with ImageJ (Version 1.53c), GraphPad Prism (Version 9.5.0), Olympus cellSens Dimension Desktop (Version 1.18), ITK-SNAP (Version 3.6.0), and G*Power (Version 3.1.9.6).

For manuscripts utilizing custom algorithms or software that are central to the research but not yet described in published literature, software must be made available to editors and reviewers. We strongly encourage code deposition in a community repository (e.g. GitHub). See the Nature Portfolio guidelines for submitting code & software for further information.

Data

Policy information about availability of data

All manuscripts must include a data availability statement. This statement should provide the following information, where applicable:

- Accession codes, unique identifiers, or web links for publicly available datasets
- A description of any restrictions on data availability
- For clinical datasets or third party data, please ensure that the statement adheres to our policy

All relevant data generated for this study are included in the article/Supplementary Information. The fluorescent images generated in this study have been deposited in the Zenodo database with the identifier [https://doi.org/10.5281/zenodo.8132255].

Human research participants

Policy information about studies involving human research participants and Sex and Gender in Research.

Reporting on sex and gender	One patient, a female, was chosen to illustrate the MRI findings of human GBM recurrence as a point of reference for the study, but was not part of a clinical trial or cohort for the manuscript.
Population characteristics	The patient (female, 64-year-old) was treated with standard of care for GBM, including surgical resection and concurrent radiation (60 Gy) and TMZ, followed by 12 monthly cycles of TMZ. At the end of treatment and for 4 years of serial MR imaging, there was no evidence of recurrence. However, within 4 months after an unchanged MR scan, the patient developed focal seizures, and a repeat MRI showed a new enhancing mass at the medial tumor margin. Since we did not choose the genes to modify based on this patient data, the patient's WHO classification and underlying tumor genetics are not relevant information for this study design.
Recruitment	The patient consented to have her MRI included in the manuscript (as per Elizabeth Maher, clinical neuro-oncologist and co-author). Under an Institutional Review Board (IRB)-approved protocol, patient with brain tumor was approached by an attending neurosurgeon or a clinical research coordinator at their initial visit to the University of Texas Southwestern Medical Center and asked to give written consent to the collection of blood and residual tissue samples in the case of surgical resection.
Ethics oversight	The study protocols using human GBM samples were performed under STU-022-011, Retrospective Studies on Clinicopathological Correlation in Neuro-Oncology. The study was approved by the Institutional Review Board (IRB), University of Texas Southwestern Medical Center. The authors state that all human experiments were performed in strict accordance with the relevant laws and institutional guidelines.

Note that full information on the approval of the study protocol must also be provided in the manuscript.

Field-specific reporting

Please select the one below that is the best fit for your research. If you are not sure, read the appropriate sections before making your selection.

☒ Life sciences ☐ Behavioural & social sciences ☐ Ecological, evolutionary & environmental sciences

For a reference copy of the document with all sections, see nature.com/documents/nr-reporting-summary-flat.pdf

Life sciences study design

All studies must disclose on these points even when the disclosure is negative.

Sample size	We used G*power analysis to calculate the sample sizes for tumor size and survival analysis. The effect size was obtained from our preliminary study. With 85% power, and alpha set to 0.05, the sample size required was calculated as n = 5 per group. The sample size is in agreement with other studies in the field, such as Yang, T., Mochida, Y., Liu, X. et al. Nat Biomed Eng 5, 1274–1287 (2021), Gregory, J.V., Kadiyala, P., Doherty, R. et al. Nat Commun 11, 5687 (2020), and Cui, Q., Yin, K., Zhang, X. et al. Nat Cancer 2, 932–949 (2021).
Data exclusions	No data was excluded from the analyses.
Replication	For in vitro experiment, six replicates were performed. For in vivo experiment, two or three independent experiments were performed. For tumor size analysis and survival rate analysis, we included five to seven animals in each group. The sample size is consistent with previously published work in the field. The number of sample size is indicated in the figure legends.
Randomization	Subject animals were randomly assigned to different experimental groups with no bias for further treatment.
Blinding	The investigators were not blinded to allocation during experiments and outcome assessment since the treatment groups were obvious from the result.

Reporting for specific materials, systems and methods

We require information from authors about some types of materials, experimental systems and methods used in many studies. Here, indicate whether each material, system or method listed is relevant to your study. If you are not sure if a list item applies to your research, read the appropriate section before selecting a response.

Materials & experimental systems

n/a	Involved in the study
☐	☒ Antibodies
☐	☒ Eukaryotic cell lines
☒	☐ Palaeontology and archaeology
☐	☒ Animals and other organisms
☒	☐ Clinical data
☒	☐ Dual use research of concern

Methods

n/a	Involved in the study
☒	☐ ChIP-seq
☒	☐ Flow cytometry
☐	☒ MRI-based neuroimaging

Antibodies

Antibodies used

1. Rat anti-CD31: Unconjugated, Clone: MEC 13.3. #550274, BD Biosciences.
2. Goat anti-CD31: Unconjugated, #AF3628, R&D Systems. (PA5-143216
3. Rat anti-transferrin: Unconjugated, Clone: 8D3, #NB100-64979, Novus Biologicals.
4. Rat anti-VEGFR2 , Unconjugated, Clone: DC101, #BE0060, Bio X Cell.
5. Rabbit anti-Claudin-5, Unconjugated, #34-1600, Invitrogen/Fisher Scientific.
6. Rabbit anti-ZO-1: Unconjugated, #402-200, Invitrogen/Fisher Scientific.
7. Rabbit anti-Occludin: Unconjugated, #71-1500, Invitrogen/Fisher Scientific.
8. Rabbit anti-VE-cadherin: Unlabeled, #36-1900, Invitrogen/Fisher Scientific.
9. Rabbit anti-ki67: Unconjugated, Clone: SP6. #MA5-14520, Invitrogen/Fisher Scientific.
10. Rat anti-JAM-A: Unconjugated, Clone BV11, produced by Dr. Monica Giannotta.
11. Rat-anti-JAM-A: Unconjugated, Clone BV12, produced by Dr. Monica Giannotta.
12. Donkey anti-goat IgG Alexa 488: Polyclonal secondary antibody, conjugated with Alexa Fluor™ 488. #A-11055, Invitrogen/Fisher Scientific.
13. Donkey anti-rat IgG Alexa 594: Polyclonal secondary antibody, conjugated with Alexa Fluor™ 594. #A-21209. Invitrogen/Fisher Scientific.
14. Donkey anti-rabbit IgG Alexa 594: Polyclonal secondary antibody, conjugated with Alexa Fluor™ 594. #A-21207. Invitrogen/Fisher Scientific.
15. Donkey anti-rabbit IgG Alexa 647: Polyclonal secondary antibody, conjugated with Alexa Fluor™ 647.# A-31573. Invitrogen/Fisher Scientific.

In general, the antibody dilution ratio was 1:500 in PBS, and 1 mL of the antibody solution was applied to 10-12 brain slices.

Validation

1. Rat anti-CD31: Reactivity: Mouse. Diluted 1:500 in PBS for IHC staining. https://www.bdbiosciences.com/content/bdb/paths/generate-tds-document.us.550274.pdf
2. Goat anti-CD31: Reactivity: Mouse, Rat. Diluted 1:500 in PBS for IHC staining. https://www.novusbio.com/products/cd31-pecam-1-antibody_af3628#datasheet
3. Rat anti-transferrin: Reactivity: Mouse. Used for nanoparticle conjugation (antibody:nanoparticle=200:1). https://www.novusbio.com/products/tfr-transferrin-r-antibody-8d3_nb100-64979#datasheet
4. Rat anti-VEGFR2: Reactivity: Mouse. Used for nanoparticle conjugation (antibody:nanoparticle=200:1). https://d2a7cdyquyl45u.cloudfront.net/tds-sheets/BE0060-tds.pdf
5. Rabbit anti-Claudin-5: Reactivity: Human, Mouse. Diluted 1:500 in PBS for IHC staining. https://www.thermofisher.com/order/genome-database/dataSheetPdf?producttype=antibody&productsubtype=antibody_primary&productId=34-1600&version=251
6. Rabbit anti-ZO-1: Reactivity: Dog, Human, Mouse, Rat. Diluted 1:500 in PBS for IHC staining. https://www.thermofisher.com/order/genome-database/dataSheetPdf?producttype=antibody&productsubtype=antibody_primary&productId=40-2200&version=251
7. Rabbit anti-Occludin: Reactivity: Dog, Human, Rat. Diluted 1:500 in PBS for IHC staining. https://www.thermofisher.com/order/genome-database/dataSheetPdf?producttype=antibody&productsubtype=antibody_primary&productId=71-1500&version=251
8. Rabbit-anti-VE-cadherin: Reactivity: Human, Mouse, Rat. Diluted 1:500 in PBS for IHC staining. https://www.thermofisher.com/order/genome-database/dataSheetPdf?producttype=antibody&productsubtype=antibody_primary&productId=36-1900&version=251
9. Rabbit anti-ki67: Reactivity: Human, Mouse, Non-human Primate, Rat. Diluted1:500 in PBS for IHC staining. https://www.thermofisher.com/order/genome-database/dataSheetPdf?producttype=antibody&productsubtype=antibody_primary&productId=MA5-14520&version=251
10. Rat anti-JAM-A (BV11): Reactivity: Mouse. Used for nanoparticle conjugation (antibody:nanoparticle=200:1). Antibody information was provided in J Cell Biol (1998) 142 (1): 117–127.
11. Rat-anti-JAM-A (BV12): Reactivity: Mouse. Diluted 1:500 in PBS for IHC staining. Antibody information was provided in J Cell Biol (1998) 142 (1): 117–127.
12. Donkey anti-goat IgG Alexa 488: Reactivity: Goat. Diluted 1:500 in PBS for IHC staining. https://www.thermofisher.com/order/genome-database/dataSheetPdf?producttype=antibody&productsubtype=antibody_secondary&productId=A-11055&version=322
13. Donkey anti-rat IgG Alexa 594: Reactivity: Rat. Diluted 1:500 in PBS for IHC staining.https://www.thermofisher.com/order/genome-database/dataSheetPdf?producttype=antibody&productsubtype=antibody_secondary&productId=A-21209&version=322
14. Donkey anti-rabbit IgG Alexa 594: Reactivity: Rabbit. Diluted 1:500 in PBS for IHC staining. https://www.thermofisher.com/order/

genome-database/dataSheetPdf?producttype=antibody&productsubtype=antibody_secondary&productId=A-21207&version=322
15. Donkey anti-rabbit IgG Alexa 647: Reactivity: Rabbit. Diluted 1:500 in PBS for IHC staining. https://www.thermofisher.com/order/genome-database/dataSheetPdf?producttype=antibody&productsubtype=antibody_secondary&productId=A-31573&version=322

Eukaryotic cell lines

Policy information about cell lines and Sex and Gender in Research

Cell line source(s)	73C and PS5A1 cell lines were established in Dr. Robert Bachoo's laboratory (Singh et al., Cell Rep. 2017, 18(4): 961–976; Gao et al., 2020, Cell Reports 30, 2489–2500).
Authentication	Both mouse cell lines were authenticated by Mouse Genome STR DNA typing PCR.
Mycoplasma contamination	All cell lines were tested negative for mycoplasma contamination.
Commonly misidentified lines (See ICLAC register)	No commonly misidentified lines were used in this study.

Animals and other research organisms

Policy information about studies involving animals; ARRIVE guidelines recommended for reporting animal research, and Sex and Gender in Research

Laboratory animals	The immunodeficient nude mice Foxn1nu (Nu/J, stock number 002019, 7 weeks old, female, 20-25 g) were ordered from Jackson Laboratories. All animals were bred in pathogen-free conditions, in temperature (20-22 degree Celcius) and humidity (52-57%)-controlled housing, under a 12-h light/dark cycle, and with free access to food and water.
Wild animals	The study did not involve wild animals.
Reporting on sex	Only female mice were used in this study.
Field-collected samples	The study did not involved samples collected from the field.
Ethics oversight	Animal protocols were approved by the Institutional Animal Care Use Committee (IACUC) of the University of Texas at Dallas.

Note that full information on the approval of the study protocol must also be provided in the manuscript.

Magnetic resonance imaging

Experimental design

Design type	The experimental procedure was provided in details in Method section under topic "Human GBM sample" and "Magnetic Resonance Imaging (MRI)".
Design specifications	For animal imaging, the MRI images were acquired before and after treatment (specifically, 3 and 15 days post injection). For human imaging, the patient received 4 years of serial MR imaging.
Behavioral performance measures	N.A.

Acquisition

Imaging type(s)	T1/T2-weighted images.
Field strength	3 T
Sequence & imaging parameters	For animal imaging, T2-weighted scans were performed using a T2 RARE sequence with an echo time of 60 ms, repetition time of 2000 ms, an echo spacing of 15 ms, a rare factor of 10, 8 averages, 1 repetition, a slice thickness of 0.5 mm, a field of view of 20 by 20 mm, and a matrix size of 192 by 192. The number of slices ranged from 12 to 20 to cover the entire brain of each mouse. F<br>The human GBM was imaged at 3T under a standard clinical brain tumor protocol that included T2/FLAIR and post-gadolinium images.
Area of acquisition	Whole brain scan.

Diffusion MRI ☐ Used ☒ Not used

Preprocessing

Preprocessing software	For animal imaging, the tumor volume was measured by manually contoured the volume of interest around the hyper-intense portion on the T2-weighted images. The data was analyzed using ImageJ (v1.53c) and ITK-SNAP (3.6.0).

Normalization	N.A.
Normalization template	N.A.
Noise and artifact removal	N.A.
Volume censoring	N.A.

Statistical modeling & inference

Model type and settings	N.A.
Effect(s) tested	N.A.

Specify type of analysis: ☐ Whole brain ☐ ROI-based ☐ Both

Statistic type for inference (See Eklund et al. 2016)	N.A.
Correction	N.A.

Models & analysis

n/a	Involved in the study
☒	☐ Functional and/or effective connectivity
☒	☐ Graph analysis
☒	☐ Multivariate modeling or predictive analysis
